# Rebuilding trust in public health: Beyond polarization

**DOI:** 10.1017/S095026882610137X

**Published:** 2026-05-21

**Authors:** Jussi Sane

**Affiliations:** Research, Development and Innovation Management, Finnish Institute for Health and Welfare, Finland

**Keywords:** Trust, public health, COVID-19, pandemic, after action review

## Abstract

The global health landscape has entered a period of profound change. Official development assistance for health has sharply declined, while new U.S. policies threaten long-standing public health norms and undermine evidence-based guidance. Vaccine hesitancy, political interference in medical recommendations, and climate policy reversals illustrate a troubling erosion of scientific authority. These developments coincide with growing polarization, revealing that ‘following the science’ is neither straightforward nor free from uncertainty. The COVID-19 pandemic exposed how public health decisions were often made under evolving evidence, with limited transparency about risks, trade-offs, and unintended consequences. Subsequent international initiatives, such as the WHO’s global agenda on Public Health and Social Measures, now seek to build a more balanced evidence base. Rebuilding public trust demands openness about uncertainty, acknowledgement of past missteps, and transparent communication of policy rationales. Structured after-action reviews and community engagement are essential to demonstrate accountability and learning. Ultimately, restoring confidence in science and public health will require humility, consistency, and cross-partisan collaboration to show that science is adaptive, responsive, and committed to improvement.

The U.S. and global health community now face a drastically changed landscape. Official development assistance for health has plunged in recent years, according to the Organisation for Economic Co-operation and Development (OECD) [1]. The Trump administration also released a new global health strategy, often referred to as the ‘America First Global Health Strategy, which, for example, replaces much of the old multilateral-focused aid model with direct bilateral agreements between the U.S. and partner countries’ [2]. At the same time, new U.S. policies threaten to roll back long-standing public health norms. For example, recent shifts in government messaging on vaccines have stoked concerns of measles resurgence [3]. Prominent officials have also publicly questioned the safety of common pregnancy medicines (e.g., warning against acetaminophen use in pregnancy), despite European Union (EU) and World Health Organization (WHO) reviews finding no evidence of harm [4]. Climate change research and mitigation programs are likewise under renewed attack, and funding for global health programs has been slashed. These moves are concerning, especially given the global influence of U.S. agencies in setting science-based standards.

These policy changes could have deep, long-lasting health impacts in the U.S. and abroad. At the same time, it is worth probing why such radical shifts are occurring. Partisan polarization certainly plays a role as health and science have become politicized across the spectrum, but the explanation may go deeper than mere ideology.

The COVID-19 era exposed that ‘following the science’ is rarely a simple, uncontroversial path. During the pandemic, many public health measures were instituted amid considerable uncertainty, often following a precautionary principle. For instance, mask mandates, school closures, and other interventions were often enacted using the rhetoric of ‘we’re following the science’, even though the evidence base was evolving [5]. This nuance was frequently lost in public discourse. In fact, global reviews note that the unintended consequences of these measures were rarely examined by early studies [6]. Only more recently is a broader research agenda taking shape: WHO has launched a global initiative to study Public Health and Social Measures (PHSM), focusing on both their effectiveness and side-effects [6]. The PHSM agenda explicitly highlights themes such as determinants of adherence and socioeconomic impacts, aiming to build a robust evidence base for future decisions [6].

Vaccination policy differences also illustrate the challenges. Throughout the pandemic and even more recently, U.S. guidance (Centers for Disease Control and Prevention, CDC) was not always aligned with international peers. For example, after the acute phase of the pandemic, European agencies like the European Center for Disease Prevention and Control (ECDC) recommended focusing boosters on older adults and high-risk groups, whereas U.S. policy continued to encourage annual boosters for all ages [7]. The European stance was and is not anti-scientific by any means. In mid-2025, the U.S. changed course: the Health Secretary announced that shots are no longer recommended for healthy children, and the CDC softened its guidance to say children may get vaccinated rather than should [8]. By contrast, bodies like the American Academy of Pediatrics immediately reaffirmed vaccinating young children as a precaution [8]. Context can and often should matter: potentially differing epidemiology, value judgements, and risk thresholds can lead to different policies, and the public notices when national guidelines conflict.

In short, we should all recall that public health is not flawless. Good-faith mistakes and simplifications were made by scientists and officials during the pandemic. We saw broad assertions like ‘we must do X because the science demands it’ even when the science was evolving and not at all settled, although the measures were probably motivated by a genuine belief that they were saving lives. Vaccination and non-pharmaceutical measures had to be decided quickly, but the inevitable uncertainties should have been transparently acknowledged. The precautionary principle should be applied cautiously and in proportion to the available evidence. Transparency about uncertainty and acknowledging errors are key to rebuilding trust [9].

Trust is the foundation of effective public health. When it erodes, people may turn to misinformation or reject vital interventions [9]. And rebuilding trust requires openness. Institutions must share the evidence and reasoning behind decisions, admit when they change course, and clearly explain why. Trust can grow again when people see that ‘science is not infallible but adaptive, responsive, and rooted in evidence’ [9]. This means publicly owning any mistakes (e.g., overzealous mandates or misestimated risks) and showing how policies will improve.

Key steps to restore confidence include acknowledging uncertainty and past missteps, being transparent and data-driven, engaging communities as partners, and conducting structured after-action reviews. Public health leaders should candidly discuss what was and was not known, emphasizing that early COVID-19 guidance was provisional and subject to revision. Explaining the limits of evidence, rather than glossing over them, helps to build credibility. Transparency is equally critical: sharing data and the rationale behind policy changes, even when uncomfortable, demonstrates that recommendations are evidence-informed and not arbitrary. Finally, systematic after-action reviews, as recommended by WHO, are vital to assess what worked, what failed, and where unintended consequences emerged [10]. These reviews should involve unbiased, independent experts, consider issues such as school closures and socioeconomic trade-offs, and, importantly, make their results public to demonstrate accountability and learning.

If the public sees only one side attacking the other, faith in science can falter. Positioning the debate purely as ‘science versus the current administration’ in any country may risk alienating people. Regaining public trust after the pandemic will require balanced self-reflection. Yes, many of the new policies (vaccine scepticism, climate rollbacks, and aid cuts) are deeply concerning. But we also must admit that health leaders and scientists sometimes oversold certainty or neglected broader harms. Building bridges means admitting these errors and committing to doing better. Trust is won through consistent transparency, not by demonizing opponents. Only by demonstrating that all stakeholders across the political spectrum are learning and improving can the population’s faith in science be restored.
